# Hardware-in-the-loop testing of CSF shunts

**DOI:** 10.1186/2045-8118-12-S1-O2

**Published:** 2015-09-18

**Authors:** Manuel Gehlen, Vartan Kurtcuoglu, Marianne Schmid Daners

**Affiliations:** 1 Department of Mechanical and Process Engineering, ETH Zurich, Switzerland; 2 Institute of Physiology, University of Zurich, Switzerland

## Introduction

Siphoning in upright posture has led to a multitude of increasingly complex cerebrospinal fluid (CSF) shunts. These shunts use a diversity of approaches to solve the problem of the resulting overdrainage. Overdrainage and the generally high failure rate of CSF shunts have led to the vision of an actively controlled shunt that adapts its drainage rate to the needs of the patient. This evolution towards more complex devices calls for fast and cost-effective in vitro testing methods that provide information on the interaction of shunt and the patient's CSF system.

## Methods

We designed a hardware-in-the-loop (HIL) test bed that provides this information by adapting the tested shunt's environment according to a real-time simulation of the patient. A mathematical model of the patient's relevant pathophysiology simulates the influence of the measured drainage through the shunt, the patient's posture, and cardiac-induced pulsations on intracranial and intraperitoneal pressure. These pressures are then applied to the proximal and distal catheter tips of the CSF shunt through highly dynamic pressure interfaces. As the posture of the simulated patient changes, the tested shunt is moved accordingly by a posture mechanism with two degrees of freedom. The resulting CSF drainage through the shunt is measured and fed back to the patient simulation in real-time.

## Results

During experiments with this HIL test bed and standard differential pressure valves we were able to replicate the problem of overdrainage: Within 9.2 ± 1.5 min after sitting up, mean ICP fell to -21.1 ± 1.3 mmHg relative to the external auditory canal due to drainage rates of up to 4.4 ± 0.2 mL/min. Avoidance of this overdrainage through the addition of a gravitational unit was also reproduced, with mean ICP in sitting position only falling to -9.1 ± 0.1 mmHg. The experiments further revealed that the pressure interfaces can reproduce even pulsatile ICP signals with less than 0.1 mmHg mean absolute error, allowing the application and analysis of any pathophysiological pulse pressure waveform. Using a 24-hour test cycle based on measured patient data allowed us to compare shunts during typical daily activities.

## Conclusion

The experiments showed that HIL testing can be used to accurately analyze and quantify the dynamic interaction between shunt and patient in a realistic yet reproducible in vitro environment. By virtue of the realistic implementation of this interaction, the results will also be valid for actively controlled shunts, which adapt to measured parameters of the patient's pathophysiology. We thus see our test bed as a catalyst for the development of future shunt systems by enabling fast and cost-effective testing of new ideas and concepts, while reducing animal trials.

